# Enzymatic Hydrolysate of Red Yeast Rice Accelerates Diabetic Wound Healing in Mice: Association with Modulation of Inflammatory, Matrix-Remodeling, and Angiogenesis-Related Markers

**DOI:** 10.3390/ijms27209020

**Published:** 2026-10-12

**Authors:** Yating Du, Weihang Ma, Xinyue Chang, Shuting Wang, Runying Zeng, Nan Wu

**Affiliations:** 1 Third Institute of Oceanography, Ministry of Natural Resources, Xiamen 361005, China; 2 Fujian Provincial Key Laboratory of Island Conservation and Development, Island Research Center, Ministry of Natural Resources, Pingtan 350400, China

**Keywords:** red yeast rice, enzymatic hydrolysis, diabetic wound healing, inflammation, angiogenesis

## Abstract

Delayed healing of diabetic wounds arises from a self-sustaining pathological microenvironment featuring persistent inflammation, excessive matrix degradation, impaired angiogenesis, and insufficient collagen deposition, and current single-process interventions remain unsatisfactory. Red yeast rice, produced by fermentation of rice with Monascus spp., is a traditional fermented product with documented anti-inflammatory and antioxidant activities; however, its direct extract is dominated by macromolecular and bound-state constituents with low release efficiency and poor skin permeation, which restricts its topical application. In this study, an enzymatic hydrolysate of red yeast rice (RYR-EH) was prepared by composite enzymatic hydrolysis, and the compositional changes induced by hydrolysis were characterized by widely targeted UPLC-MS/MS metabolomics. A total of 810 metabolites were detected, among which 199 showed differential accumulation after hydrolysis (179 upregulated and 20 downregulated; FC ≥ 2 or ≤0.5, VIP ≥ 1, and *p* < 0.05); the differential metabolites were significantly enriched (FDR < 0.05) in purine metabolism, nucleotide metabolism, ABC transporters, and the cGMP-PKG signaling pathway, a pattern consistent with hydrolysis-driven release of free amino acids, small peptides, and other low-molecular-weight constituents. In type 2 diabetic mice induced by a high-fat diet combined with low-dose streptozotocin, topical RYR-EH (2% and 5%) significantly accelerated the closure of full-thickness excisional wounds, with the 5% formulation showing more advanced closure than the 2% formulation and an effect approaching that of recombinant human epidermal growth factor (rhEGF). At the day-14 endpoint, RYR-EH treatment was associated with enhanced re-epithelialization, lower TNF-α, IL-6, and IL-1β levels in both wound tissue and serum, reduced MMP-2 and MMP-9 immunoreactivity, reduced Ki67-positive cellularity in the late-stage wound bed, increased Collagen I/III deposition, and increased VEGF/CD31 immunoreactivity. These results demonstrate that RYR-EH exerts multi-process reparative effects on diabetic wounds, simultaneously attenuating inflammation, restraining excessive matrix degradation, promoting angiogenesis, and restoring collagen deposition, and support RYR-EH as an effective multi-process topical agent for diabetic wound healing.

## 1. Introduction

Diabetic wounds, represented by diabetic foot ulcers, are among the most common and severe chronic complications of diabetes mellitus and a leading cause of lower-limb amputation, imposing a substantial clinical and economic burden worldwide [1]. Unlike acute wounds, diabetic wounds are trapped in a self-sustaining pathological microenvironment. Sustained hyperglycemia and oxidative stress keep the wound locked in the inflammatory phase, with persistently elevated pro-inflammatory cytokines such as tumor necrosis factor-α (TNF-α), interleukin-6 (IL-6), and IL-1β [2,3,4]. Matrix metalloproteinases (MMP-2 and MMP-9) are overactivated and continuously degrade the extracellular matrix (ECM) and nascent granulation tissue [5,6]. Meanwhile, vascular endothelial growth factor (VEGF)-mediated angiogenesis is blunted, leaving the wound bed hypoperfused, and fibroblast proliferation, collagen deposition, and keratinocyte-driven re-epithelialization are all impaired [7,8]. Because these pathological events reinforce one another, current clinical management—including debridement, passive dressings, negative-pressure therapy, and recombinant growth factors such as epidermal growth factor (EGF)—acts largely on a single process, and the therapeutic outcomes remain unsatisfactory, with high recurrence rates and considerable cost [9,10]. Therefore, well-tolerated and cost-effective agents capable of coordinately modulating multiple pathological processes of the diabetic wound are urgently needed.

Food-derived natural products have attracted increasing attention in chronic wound management because of their structural diversity, multi-process potential, and favorable safety profiles [11]. Red yeast rice (RYR), produced by fermentation of rice with *Monascus* spp., is a traditional fermented product with a long history of dietary and medicinal use in East Asia. It contains *Monascus* pigments, monacolins, polysaccharides, flavonoids, and phenolic compounds, and its anti-inflammatory, antioxidant, and metabolic regulatory activities have been well documented [12,13,14]. Despite these attributes, the application of red yeast rice in skin wound repair, particularly in diabetic wounds, has not been reported. A practical limitation underlies this gap: in directly prepared water or ethanol extracts, the majority of bioactive constituents exist in macromolecular or bound forms embedded in the cereal cell-wall matrix, resulting in low release efficiency, high molecular weight, and poor skin permeation, which severely restricts the topical efficacy of the raw extract.

Degradation or modification of raw materials is a widely adopted strategy to release bound-state constituents and enhance the bioactivities of food-derived ingredients. Among the available approaches, physical degradation often suffers from incomplete and unstable outcomes, whereas chemical degradation risks environmental pollution and product contamination. In contrast, enzymatic hydrolysis offers mild reaction conditions, environmental friendliness, and stable product quality, and has been extensively applied to cereals and medicinal fungi to improve the release and bioavailability of their active components [15,16]. For red yeast rice, composite enzymatic hydrolysis with cellulase, amylase, and protease can disrupt the cell-wall architecture of the fermented grain, degrade macromolecular starch and protein, and liberate bound small molecules, thereby converting the raw material into a preparation enriched in low-molecular-weight, readily absorbable constituents. Widely targeted metabolomics based on UPLC-MS/MS provides a robust tool to verify such conversion at the compositional level, enabling quantitative comparison of metabolite profiles before and after hydrolysis and mapping of differential metabolites onto functional pathways [17]. To date, however, no study has integrated enzymatic hydrolysis of red yeast rice with metabolomic profiling and in vivo evaluation in the context of diabetic wound repair.

Based on these considerations, we hypothesized that enzymatic hydrolysis of red yeast rice would generate a hydrolysate with a remodeled, repair-relevant metabolite profile and superior wound-healing efficacy compared with the unhydrolyzed extract. Accordingly, in the present study, the enzymatic hydrolysate of red yeast rice (RYR-EH) was prepared using a composite enzyme system, and hydrolysis-induced compositional changes were characterized by widely targeted metabolomics. The therapeutic efficacy of topically applied RYR-EH was then evaluated in a type 2 diabetic mouse model induced by a high-fat diet combined with low-dose streptozotocin (STZ), using full-thickness excisional wounds and combining gross wound-closure kinetics with histopathological and molecular analyses covering inflammation (TNF-α, IL-6, IL-1β), matrix turnover (MMP-2, MMP-9, Collagen I, Collagen III), cell proliferation (Ki67), and angiogenesis-related markers (VEGF/CD31). Because a direct in vivo comparison between RYR-EH and the unhydrolyzed extract was not included in the present experiment, statements attributing the observed efficacy to enzymatic hydrolysis are formulated as a hypothesis and are interpreted with corresponding caution throughout this manuscript.

## 2. Results

### 2.1. Enzymatic Hydrolysis Reshapes the Metabolite Profile of Red Yeast Rice and Enriches Repair-Relevant Pathways

**Figure 1:**
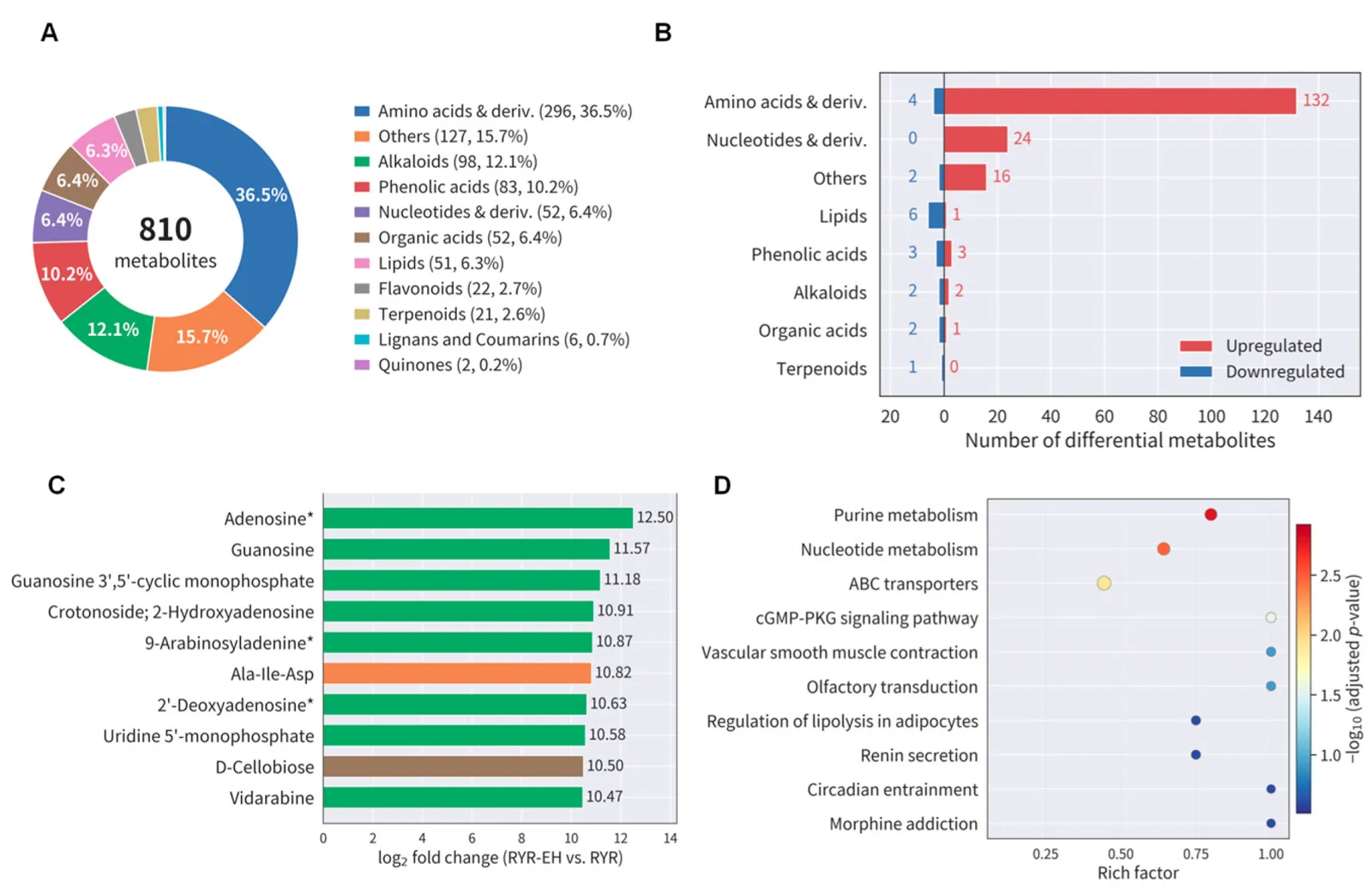
Widely targeted metabolomic analysis of red yeast rice before (RYR) and after enzymatic hydrolysis (RYR-EH). (**A**) Chemical classification of the 810 detected metabolites; (**B**) numbers of upregulated and downregulated differential metabolites in each chemical class (RYR-EH vs. RYR; screened by fold change FC ≥ 2 or ≤0.5, VIP ≥ 1, and *p* < 0.05 with FDR adjustment); (**C**) top 10 upregulated differential metabolites ranked by log2 fold change, colored by chemical class; (**D**) KEGG pathway enrichment bubble plot of the differential metabolites (top 10 pathways ranked by adjusted *p* value, with the generic KEGG overview map ‘Metabolic pathways’ (ko01100) omitted for clarity; bubble size indicates the number of enriched metabolites; color indicates the adjusted *p* value). Raw and adjusted *p* values and mapped metabolite counts per pathway are provided in Table S3. In (**C**), an asterisk (*) denotes an annotation flagged by the metabolomics database (Metware Biotech) as an isomer that cannot be unambiguously distinguished from its structural isomer(s) by MS/MS alone; the named compound represents the best-matching structure.

Widely targeted UPLC-MS/MS-based metabolomic analysis was performed to characterize the compositional remodeling of red yeast rice induced by enzymatic hydrolysis. A total of 810 metabolites were detected across the unhydrolyzed red yeast rice extract (RYR) and the enzymatic hydrolysate (RYR-EH) samples, which were assigned to 11 chemical classes dominated by amino acids and derivatives (296), alkaloids (98), phenolic acids (83), nucleotides and derivatives (52), organic acids (52), and lipids (51) (Figure 1A). Principal component analysis showed clear separation of the RYR and RYR-EH groups along PC1 (74.4%) and tight clustering of the pooled quality-control samples, supporting the analytical reproducibility of the profiling (Figure S3). Applying the combined criteria of fold change (FC ≥ 2 or ≤ 0.5), VIP ≥ 1 from OPLS-DA, and *p* < 0.05 from Student’s t-test with Benjamini–Hochberg false-discovery-rate adjustment (see Section 4.3), 199 differentially accumulated metabolites were identified between RYR-EH and RYR, including 179 upregulated and 20 downregulated metabolites (Figure 1B). These 199 differentially accumulated metabolites, together with their identification confidence levels (Level 1–3), fold changes, VIP scores, exact *p* values, FDR values, and KEGG identifiers, are listed in Table S2. The upregulated metabolites were dominated by amino acids and derivatives (132 of 179) and nucleotides and derivatives, whereas lipids were predominantly downregulated (6 of the 7 lipid DAMs, accounting for 6 of the 20 downregulated metabolites). The top 10 upregulated metabolites showed log2 fold changes of 10.47–12.50 (approximately 1400- to 5800-fold increases) and were headed by nucleosides and nucleotide derivatives such as adenosine, guanosine, and 2′-deoxyadenosine, together with small peptides and oligosaccharides (Figure 1C). KEGG pathway enrichment analysis (hypergeometric test against the 197 KEGG-annotated metabolites among the 810 detected as the background set, with Benjamini–Hochberg FDR adjustment) showed that the differential metabolites were significantly enriched (FDR < 0.05) in purine metabolism, nucleotide metabolism, ABC transporters, and the cGMP-PKG signaling pathway (Figure 1D; raw and adjusted *p* values, and the number of mapped metabolites per pathway, are provided in Table S3). These annotation results describe the compositional changes in the preparation; they do not by themselves establish that the annotated metabolites are bioavailable or biologically active at the wound site. Collectively, these results indicate that enzymatic hydrolysis substantially reshaped the metabolite profile of red yeast rice, markedly increasing the abundance of amino acid- and nucleotide-related metabolites that are potentially relevant to tissue repair processes.

### 2.2. RYR-EH Accelerates Wound Closure in Diabetic Mice

**Figure 2:**
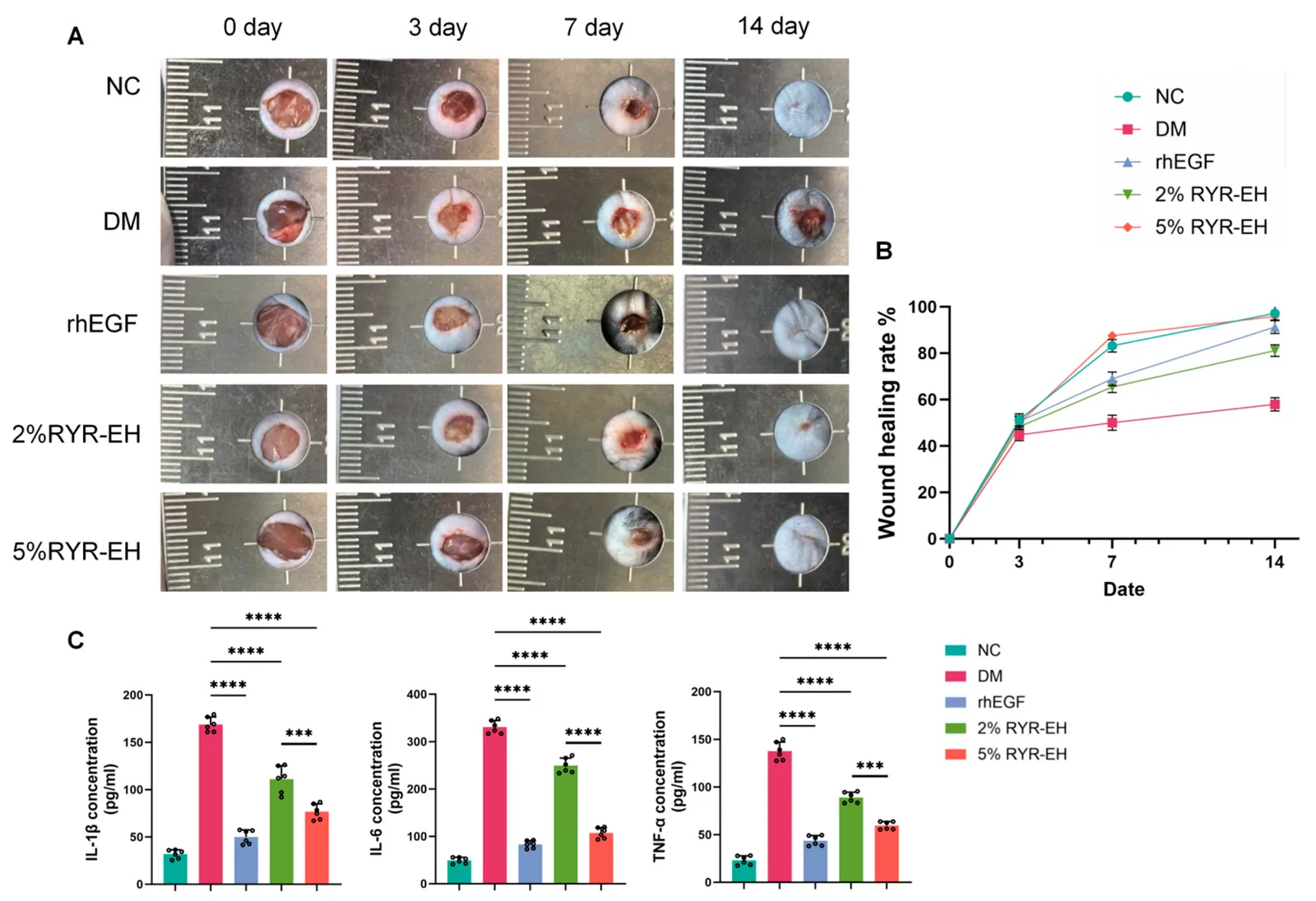
RYR-EH accelerates the closure of full-thickness wounds in diabetic mice. (**A**) Representative wound images on days 0, 3, 7, and 14; (**B**) wound closure rate over time; (**C**) serum levels of IL-1β, IL-6, and TNF-α measured by ELISA at day 14. NC: normal control; DM: diabetic model; rhEGF: recombinant human epidermal growth factor positive control; 2% RYR-EH and 5% RYR-EH: wounds treated with the 2% and 5% RYR-EH formulations, respectively. Data are presented as mean ± SD with individual data points (*n* = 6). Statistical analysis: two-way repeated-measures ANOVA with Tukey’s post hoc test (**B**); one-way ANOVA with Tukey’s post hoc test (**C**). *** *p* < 0.001, **** *p* < 0.0001.

Compared with the normal control (NC) group, diabetic model (DM) mice exhibited markedly delayed wound closure throughout the observation period (Figure 2A,B). RYR-EH treatment accelerated wound closure, with an improving trend observable from day 3 and a significant difference relative to the DM group from day 7 onward (group × time interaction, two-way repeated-measures ANOVA, *p* < 0.0001). At day 14, wounds in the 5% RYR-EH group were nearly completely closed, and wound closure in the 5% RYR-EH group was more advanced than that in the 2% RYR-EH group (*p* < 0.001), approaching the closure observed in the recombinant human epidermal growth factor (rhEGF) positive control group. In addition, serum ELISA revealed markedly elevated levels of the pro-inflammatory cytokines IL-1β, IL-6, and TNF-α in DM mice, whereas RYR-EH treatment reduced these cytokine levels (*p* < 0.0001; Figure 2C), with lower levels in the 5% RYR-EH group than in the 2% RYR-EH group (*p* < 0.001) and values approaching those of the rhEGF group. Fasting blood glucose and body weight were monitored throughout modeling and treatment; the four diabetic groups remained comparably hyperglycemic throughout the experiment, with no significant differences among the four diabetic groups in fasting blood glucose or body weight (Figure S1), indicating that differences in metabolic severity did not confound the wound-healing comparison. These results indicated that RYR-EH promoted the closure of diabetic full-thickness wounds and was associated with reduced systemic levels of the pro-inflammatory cytokines IL-1β, IL-6, and TNF-α.

### 2.3. RYR-EH Promotes Re-Epithelialization and Collagen Deposition

**Figure 3:**
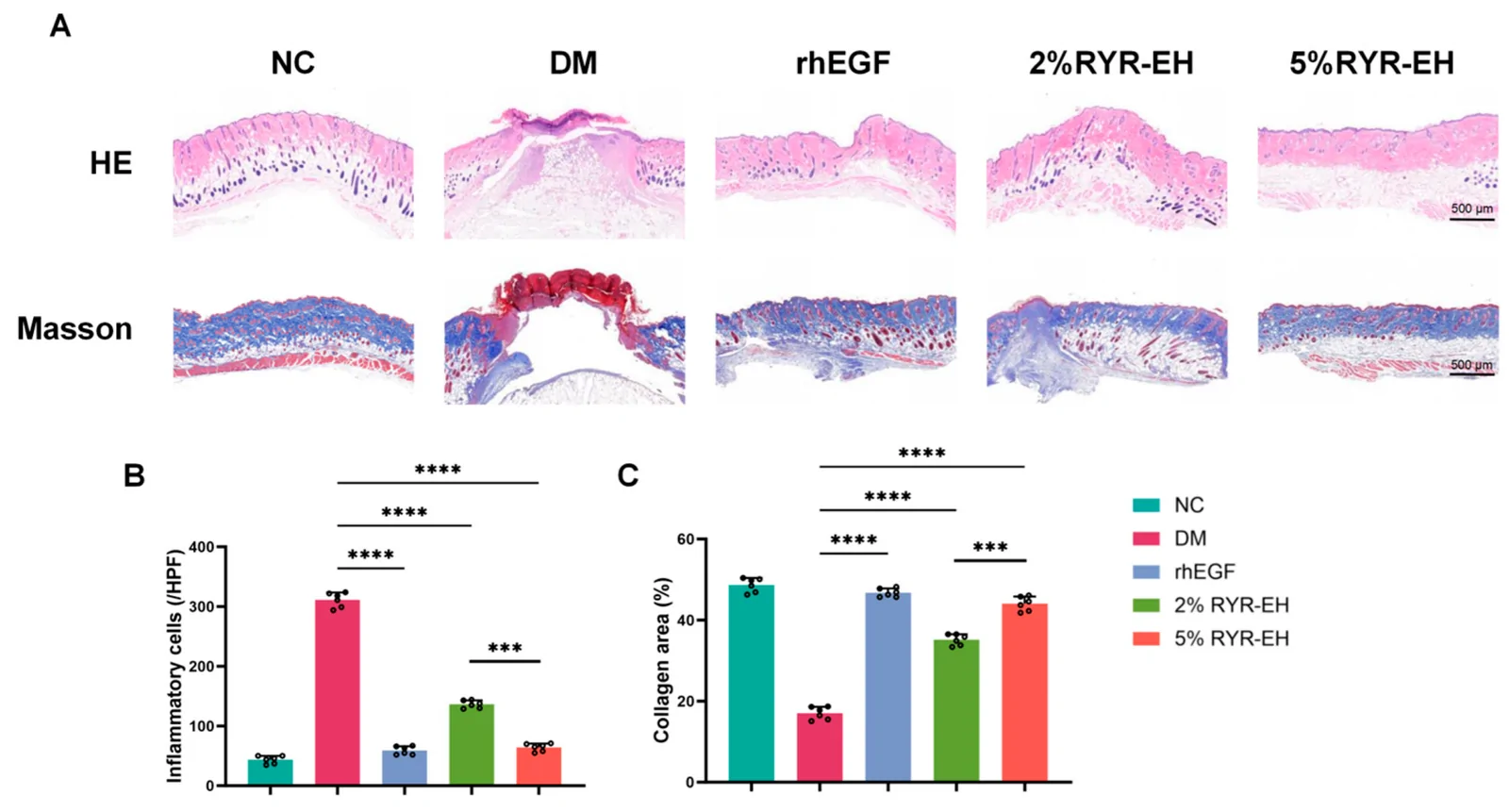
Histological analysis of wound tissue at day 14. (**A**) Representative H&E- and Masson trichrome-stained wound sections (scale bars: 500 μm); (**B**) quantification of infiltrating inflammatory cells per high-power field (HPF); (**C**) quantification of the collagen area fraction. Data are presented as mean ± SD with individual data points (*n* = 6). One-way ANOVA with Tukey’s post hoc test. *** *p* < 0.001, **** *p* < 0.0001.

H&E staining of wound sections collected at day 14 showed that re-epithelialization was essentially complete in the RYR-EH-treated groups, whereas the DM group still displayed incomplete epidermal coverage and persistent inflammatory infiltration (Figure 3A). Quantitative analysis confirmed that infiltrating inflammatory cells accumulated markedly in the wounds of the DM group compared with the NC group, and that this accumulation was reduced by RYR-EH treatment (*p* < 0.0001; Figure 3B), with lower cell numbers in the 5% RYR-EH group than in the 2% RYR-EH group (*p* < 0.001) and values comparable to those of the rhEGF group. Masson trichrome staining further showed that collagen fibers in the RYR-EH-treated wounds were denser and more regularly aligned than those in the DM group (Figure 3A), and the collagen area fraction, which was markedly reduced in the DM group, was increased by RYR-EH treatment (*p* < 0.0001; Figure 3C), with the 5% RYR-EH group approaching the levels of the NC and rhEGF groups and exceeding that of the 2% RYR-EH group (*p* < 0.001). At the day-14 endpoint, these findings indicated that RYR-EH treatment was associated with more advanced re-epithelialization, less inflammatory cell infiltration, and increased organized collagen deposition in diabetic wounds; because these endpoints were measured at a single late time point, they are interpreted as associations rather than temporally resolved mechanisms.

### 2.4. RYR-EH Attenuates Inflammatory Cytokine Expression and Is Associated with Reduced Ki67-Positive Cellularity at the Late Repair Stage

**Figure 4:**
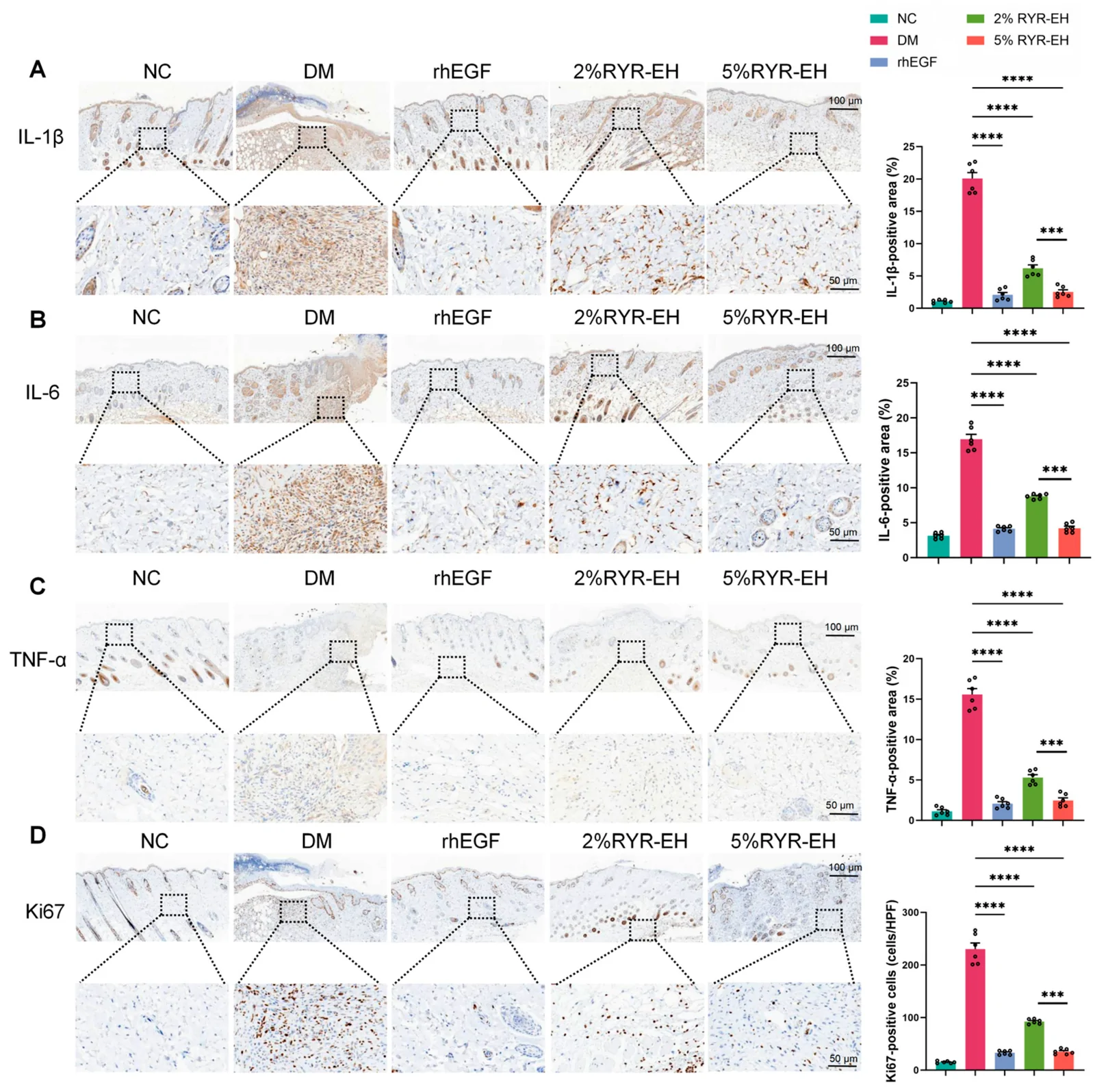
Immunohistochemical analysis of wound tissue at day 14. (**A**–**C**) Representative images and quantification of IL-1β (**A**), IL-6 (**B**), and TNF-α (**C**) positive areas; (**D**) representative images and quantification of Ki67-positive cells per HPF. Scale bars: 100 μm (upper rows) and 50 μm (magnified views). Data are presented as mean ± SD with individual data points (*n* = 6). One-way ANOVA with Tukey’s post hoc test. *** *p* < 0.001, **** *p* < 0.0001.

Immunohistochemical analysis of wound sections at day 14 showed that the pro-inflammatory cytokines IL-1β, IL-6, and TNF-α were strongly upregulated in the wounds of the DM group compared with the NC group, and their expression was reduced by RYR-EH treatment (*p* < 0.0001; Figure 4A–C), with lower expression in the 5% RYR-EH group than in the 2% RYR-EH group (*p* < 0.001) and values comparable to those of the rhEGF group. In addition, at the late repair stage (day 14), Ki67-positive cells remained persistently present in large numbers in the wound bed of the DM group, whereas their number was markedly lower in the RYR-EH-treated groups (*p* < 0.0001; Figure 4D), with the 5% RYR-EH group approaching the levels of the rhEGF and NC groups, whose wounds were already closed or nearly closed. Because Ki67 is a general proliferation marker expressed by multiple cell populations, and because only a single late time point was examined, this finding was interpreted neutrally as reduced late-stage cellular proliferation in RYR-EH-treated wounds, without implying that this reduction mediates healing or distinguishes pathological from physiological proliferating cell types. Together, at the day-14 endpoint, these results indicated that RYR-EH treatment was associated with attenuated local inflammatory cytokine expression and with reduced Ki67-positive cellularity in the late-stage wound bed of diabetic mice.

### 2.5. RYR-EH Treatment Is Associated with Reduced MMP-2/MMP-9 Immunoreactivity and Increased VEGF/CD31 Immunoreactivity in Diabetic Wounds

**Figure 5:**
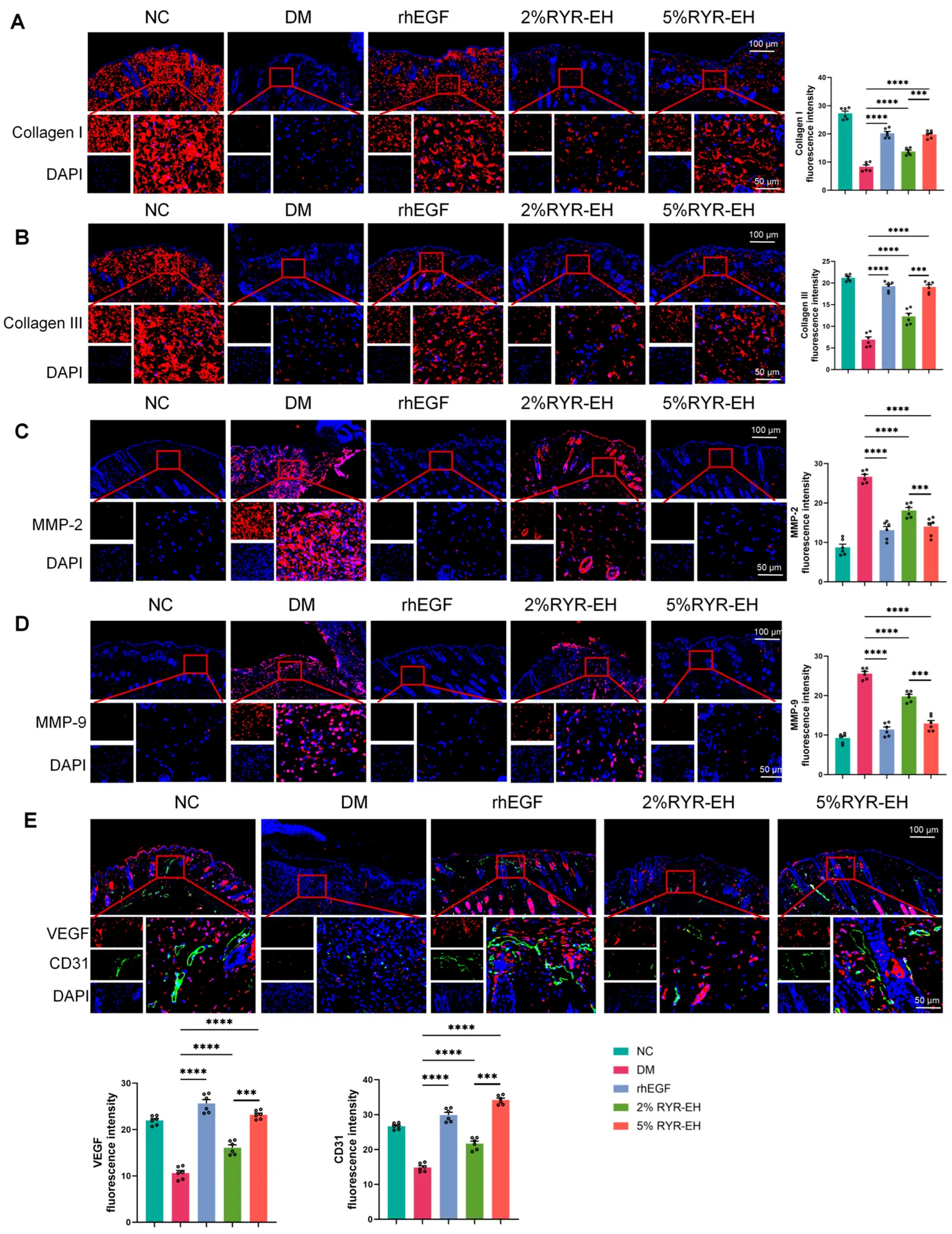
Immunofluorescence analysis of wound tissue at day 14. (**A**,**B**) Collagen I (**A**) and Collagen III (**B**) staining (red) with DAPI counterstaining (blue); (**C**,**D**) MMP-2 (**C**) and MMP-9 (**D**) staining (red) with DAPI counterstaining (blue); (**E**) double immunofluorescence staining for VEGF (red) and CD31 (green) with DAPI counterstaining (blue). Scale bars: 100 μm (whole-section views) and 50 μm (magnified views). Mean fluorescence intensity was quantified from five random fields per section. Data are presented as mean ± SD with individual data points (*n* = 6). One-way ANOVA with Tukey’s post hoc test. *** *p* < 0.001, **** *p* < 0.0001.

Immunofluorescence staining of wound sections at day 14 showed that Collagen I and Collagen III deposition was increased in RYR-EH-treated wounds compared with the DM wounds (Figure 5A,B), whereas MMP-2 and MMP-9 immunoreactivity was reduced (Figure 5C,D). Double immunofluorescence staining for VEGF and CD31 further revealed more VEGF/CD31 double-positive structures in the RYR-EH-treated wound beds (*p* < 0.0001; Figure 5E). Quantitative analysis confirmed that the fluorescence intensities of Collagen I and Collagen III were significantly increased, whereas those of MMP-2 and MMP-9 were significantly reduced, in RYR-EH-treated wounds compared with the DM group (*p* < 0.0001), with more pronounced changes at the 5% concentration than at the 2% concentration (*p* < 0.001) and values approaching those of the rhEGF group. Because immunofluorescence intensity reflects protein abundance rather than enzymatic activity, the MMP-2/MMP-9 findings were interpreted as reduced immunoreactivity rather than as direct evidence of reduced matrix degradation, and the VEGF/CD31 findings were interpreted as increased expression of angiogenesis-associated markers rather than as demonstrated formation of functional, perfused vessels. At the day-14 endpoint, RYR-EH treatment was thus associated with increased collagen deposition, reduced MMP-2/MMP-9 immunoreactivity, and increased VEGF/CD31 immunoreactivity in diabetic wounds.

## 3. Discussion

In the present study, we showed that the enzymatic hydrolysate of red yeast rice significantly accelerated the healing of diabetic full-thickness wounds in mice. At the day-14 endpoint, RYR-EH treatment was associated with attenuated local and systemic inflammation (reduced TNF-α, IL-6, and IL-1β in wound tissue and serum), reduced MMP-2 and MMP-9 immunoreactivity, increased VEGF/CD31 immunoreactivity, and increased collagen deposition together with reduced Ki67-positive cellularity in the late-stage wound bed, coinciding with accelerated re-epithelialization and wound closure. The high-dose RYR-EH formulation achieved wound closure approaching that of the rhEGF positive control, a clinically used growth factor [10]. Because all histological and molecular endpoints were measured at a single late time point, these endpoint associations cannot by themselves establish the causal sequence of healing, and the coordinated mode of action outlined above should be regarded as a consistent interpretive framework supported by parallel endpoint changes rather than as a demonstrated mechanism.

The metabolomic comparison before and after hydrolysis provides a compositional context for these findings. Enzymatic hydrolysis systematically remodeled the small-molecule profile of red yeast rice, with 179 metabolites showing increased abundance and the top 10 increasing by up to approximately 5800-fold, and the differential metabolites were significantly enriched in purine metabolism, nucleotide metabolism, ABC transporters, and the cGMP-PKG signaling pathway (FDR < 0.05; Table S3). We emphasize that pathway annotation describes the compositional changes in the preparation and does not establish that the annotated metabolites are bioavailable, reach relevant wound cells, or mediate the healing response; the connection between this repair-relevant compositional pattern and the in vivo outcomes is therefore correlative and hypothesis-generating. Within this framework, the prominence of amino acids and derivatives among the upregulated metabolites (132 of 179) is consistent with the provision of substrates for collagen biosynthesis, nitric oxide-dependent angiogenesis, and glutathione synthesis [18,19,20], paralleling the enhanced collagen deposition, increased VEGF/CD31 immunoreactivity, and reduced pro-inflammatory cytokines observed at the day-14 endpoint. Meanwhile, the significant enrichment of ABC transporter-related metabolites is consistent with hydrolysis-driven release of low-molecular-weight constituents, addressing in principle the poor release efficiency of the unhydrolyzed extract. Similar enhancement of bioactivity following enzymatic degradation has been reported for other food-derived materials [15,16]. We therefore hypothesize that enzymatic hydrolysis acts as a key transformation that generates a repair-oriented metabolite repertoire; however, because the in vivo experiment did not include an unhydrolyzed red yeast rice group at an equivalent dose, this hypothesis remains to be tested in a direct head-to-head comparison, which is underway in our laboratory.

Although this is, to our knowledge, the first report of an enzymatic hydrolysate of red yeast rice for skin wound repair, enzymatic hydrolysates from other biological sources have shown related reparative activities, which allows a horizontal comparison. Bioactive collagen peptides obtained by enzymatic hydrolysis of fish skin have been shown to enhance cutaneous repair by reprogramming the wound microenvironment toward an antioxidant, anti-inflammatory, and pro-remodeling state [21], and oral supplementation with hydrolyzed collagen improved skin elasticity and dermal matrix density in randomized, placebo-controlled trials [22]. Beyond protein-derived hydrolysates, multicomponent natural topical preparations such as medical-grade honey have shown benefit for diabetic foot ulcers in a systematic review and meta-analysis of randomized controlled trials [23]. Compared with these agents, RYR-EH is distinct in that it derives from a fermented cereal matrix rather than animal collagen and comprises a chemically diverse repertoire of free amino acids, small peptides, and nucleotide-related metabolites rather than a peptide-only fraction; moreover, RYR-EH was applied topically and acted directly on the wound bed, achieving high local exposure without requiring systemic absorption. RYR-EH therefore broadens the range of enzymatic hydrolysates available for topical wound management, which is particularly relevant for diabetic wounds in which a single active principle is unlikely to be sufficient.

The wound-healing activity of RYR-EH is also coherent with the established pharmacological profile of red yeast rice itself, which provides a vertical analogy. Beyond the cholesterol-lowering activity of monacolin K [13], red yeast rice preparations have demonstrated cardiovascular benefit in large randomized trials, including the China Coronary Secondary Prevention Study [24], and a subgroup analysis of this trial showed a reduction in cardiovascular events specifically in patients with type 2 diabetes [25]; meta-analyses support a favorable efficacy and safety profile [26]. More directly relevant to the present findings, the Monascus-derived azaphilone pigments monascin and ankaflavin exert anti-inflammatory and antioxidant effects in vivo [27], consistent with the reduced pro-inflammatory cytokines in RYR-EH-treated wounds. Enzymatic hydrolysis thus extends the pharmacological spectrum of red yeast rice from systemic metabolic and anti-inflammatory effects to topical tissue repair.

Another notable feature of the present findings is the coherence between the metabolomic profile and the in vivo efficacy. The constituents enriched by hydrolysis, including free amino acids, small peptides, and nucleotide-related compounds, map onto pathways implicated in wound repair, and this pronounced compositional remodeling was accompanied by parallel modulation of inflammation-, matrix-, and angiogenesis-related endpoints in diabetic wounds. Such a multimodal endpoint profile would be well suited to diabetic wounds, in which inflammation, matrix degradation, and vascular insufficiency form a self-sustaining pathological network that single-process interventions cannot efficiently interrupt [9,28]. The wide availability of red yeast rice as a raw material further supports the preclinical investigation of RYR-EH for chronic wound management. At the same time, dietary use does not establish the safety of topical application to open wounds, and the standardization and cost of RYR-EH were not analysed in this study; these attributes require dedicated evaluation before any translational claim can be made.

Several limitations of this study should be noted. First, the in vivo experiment did not include an unhydrolyzed red yeast rice group at an equivalent dose; consequently, the contribution of enzymatic hydrolysis per se to the observed healing effect could not be isolated, and statements attributing efficacy to hydrolysis should be read as a hypothesis. Second, all histological and molecular endpoints were measured at a single late time point (day 14); earlier repair stages were not examined, so the findings represent endpoint associations rather than temporally resolved mechanisms, and the lower inflammatory markers, reduced Ki67-positive cellularity, and altered matrix- and vascular-related signals observed in treated wounds may partly reflect more advanced healing rather than cause it. Third, Ki67 is a general proliferation marker, and the cell types contributing to the Ki67 signal were not identified; the finding was therefore interpreted neutrally as reduced late-stage cellular proliferation, and cell type-specific co-staining (e.g., with keratinocyte, fibroblast, endothelial, or macrophage markers) and dynamic evaluation during the proliferative phase will be required. Fourth, microvessel functionality was not assessed: VEGF/CD31 double immunofluorescence reflects marker expression rather than the formation of mature, perfused vessels, and perivascular markers (e.g., α-SMA or NG2) and perfusion measurements were not included. Fifth, MMP-2/MMP-9 immunoreactivity does not necessarily reflect enzymatic activity, because MMPs can be present as inactive pro-forms; gelatin zymography was not performed, and collagen deposition was assessed without evaluating collagen maturity, crosslinking, or mechanical quality. Sixth, the excisional wounds were not splinted, so healing proceeded partly through wound contraction, which differs from the granulation- and re-epithelialization-dependent repair of human diabetic ulcers. Seventh, only male mice were used, and the findings may not generalize across sexes; validation in female animals is warranted. Eighth, the percutaneous absorption and local pharmacokinetics of RYR-EH were not determined, and the rheological properties, release behaviour, and batch-to-batch consistency of the formulations were not characterized. Ninth, red yeast rice can contain pharmacologically active monacolins and the mycotoxin citrinin; the monacolin K and citrinin contents of RYR-EH were not quantified in this study, and local tolerability was not systematically assessed, so the safety of RYR-EH on open diabetic wounds remains to be established. Finally, although widely targeted metabolomics delineated the differential metabolite repertoire and repair-related pathways, the individual bioactive constituents responsible for the healing effect remain to be isolated and verified, and their structure–activity relationships and potential synergies warrant further investigation. Ongoing studies in our laboratory are addressing these issues, including a head-to-head comparison with the unhydrolyzed extract, earlier time point analyses, cell type-specific co-staining, MMP activity assays, skin-permeation studies, and contaminant quantification.

## 4. Materials and Methods

### 4.1. Materials and Reagents

Red yeast rice was obtained from Wuhan Jiacheng Biological Products Co., Ltd. (Wuhan, China). Cellulase (from Trichoderma reesei; activity 11,800 U/g), α-amylase (from Bacillus licheniformis; activity 4000 U/g), and neutral protease (from Bacillus subtilis; activity 50,000 U/g) were purchased from Shanghai Aladdin Biochemical Technology Co., Ltd. (Shanghai, China). The high-fat diet (60% kcal from fat; D12492) was supplied by Research Diets (New Brunswick, NJ, USA). Streptozotocin (STZ) was purchased from Sigma-Aldrich (St. Louis, MO, USA), and recombinant human epidermal growth factor (rhEGF) from PeproTech (Cranbury, NJ, USA). ELISA kits for TNF-α, IL-6, and IL-1β were obtained from R&D Systems (Minneapolis, MN, USA). Antibodies against TNF-α, IL-6, IL-1β, Ki67, MMP-2, MMP-9, Collagen I, Collagen III, VEGF, and CD31 were provided by Hunan Aifang Biotechnology Co., Ltd. (Changsha, China); their host species, clonality, catalogue numbers, and working dilutions are listed in Table S1, and all were used at a dilution of 1:200. All other reagents were of analytical grade.

### 4.2. Preparation of RYR-EH

RYR-EH was prepared by composite enzymatic hydrolysis following established procedures with minor modifications [15,16]. Briefly, red yeast rice was milled, passed through a 40-mesh sieve, mixed with distilled water at a solid-to-liquid ratio of 1:10 (*w*/*v*), and sterilized at 100 °C for 15 min. After cooling to room temperature, the slurry was adjusted to pH 6.0 and hydrolyzed with a composite enzyme system consisting of cellulase (2%, *w*/*w* of substrate), α-amylase (1%), and neutral protease (1%) at 50 °C for 4 h under continuous stirring. After hydrolysis, the enzymes were inactivated by heating at 100 °C for 15 min, and the hydrolysate was centrifuged at 10,000 rpm for 10 min. The supernatant was collected, concentrated under reduced pressure, and freeze-dried to obtain RYR-EH powder. The degree of hydrolysis was 28.6%, the yield of freeze-dried RYR-EH was 32.5% (*w*/*w* of starting red yeast rice), and the peptide, free amino acid, and total phenolic contents of RYR-EH were 12.4 mg/g, 8.7 mg/g, and 6.3 mg gallic acid equivalents/g, respectively (measurement methods as in [15,16]). The unhydrolyzed red yeast rice preparation (RYR) was prepared in parallel under identical conditions without the enzymatic step. Throughout this manuscript, “red yeast rice” denotes the raw material, “RYR” denotes the unhydrolyzed aqueous preparation and its freeze-dried extract, and “RYR-EH” denotes the composite enzymatic hydrolysate and its freeze-dried powder; the overall preparation scheme is shown in Figure S2. For topical application, the freeze-dried RYR-EH powder was incorporated into a carbomer 940 hydrogel base at 2% or 5% (*w*/*w*, relative to the total mass of the formulation): carbomer 940 (1%, *w*/*v*) was fully swollen in distilled water overnight, neutralized to pH 6.5–7.0 with triethanolamine, and mixed with glycerol (5%, *v*/*v*), after which RYR-EH was added and homogenized. The hydrogel base without RYR-EH served as the vehicle control. Approximately 100 mg of the corresponding formulation was applied to each wound once daily from day 0 to day 14. The viscosity, release behaviour, and batch-to-batch consistency of the formulations were not characterized in this study and are noted as limitations.

### 4.3. Widely Targeted Metabolomic Analysis

Metabolite profiling of RYR-EH and RYR was performed by Metware Biotechnology Co., Ltd. (Wuhan, China) using a UPLC-MS/MS platform (ExionLC AD coupled with a triple-quadrupole mass spectrometer; SCIEX, Framingham, MA, USA) operated in multiple reaction monitoring (MRM) mode. Each group comprised three independent biological replicates (preparations). Metabolites were annotated against the Metware database (MWDB); the 810 reported metabolites represent the union of metabolites annotated across all samples. Quality-control (QC) samples were prepared by pooling equal aliquots of all samples and were analysed at regular intervals throughout the run to monitor instrument stability; metabolites with a coefficient of variation in peak area > 30% in QC samples were excluded from further analysis. Peak integration and annotation were performed using the Metware in-house data-processing workflow. Data were log-transformed and auto-scaled (Pareto scaling) prior to multivariate analysis. Differentially accumulated metabolites were screened using fold change (FC ≥ 2 or ≤ 0.5) combined with a variable importance in projection (VIP) score ≥ 1 from orthogonal partial least squares discriminant analysis (OPLS-DA) and *p* < 0.05 from Student’s t-test with Benjamini–Hochberg false-discovery-rate (FDR) adjustment. All differential metabolites, with fold changes, VIP scores, exact *p* values, FDR values, identification confidence, and KEGG identifiers, are provided in Table S2. KEGG pathway enrichment analysis of the differential metabolites was performed using a hypergeometric test with all KEGG-annotated metabolites detected in this study (197 of the 810 detected metabolites) as the background (reference) set; duplicated or ambiguously mapped metabolites were counted once per pathway, and pathway *p* values were adjusted by the Benjamini–Hochberg method, with an FDR threshold of 0.05 considered significant. Principal component analysis (PCA) of all samples and QC samples is shown in Figure S3.

### 4.4. Animals, Diabetic Model, and Wounding

Male C57BL/6 mice (6–8 weeks old, SPF grade) were purchased from Shanghai SLAC Laboratory Animal Co., Ltd. (Shanghai, China) and acclimatized for one week. Only male mice were used to reduce biological variability, because oestrogen status is known to influence wound-healing kinetics; accordingly, the findings of this study cannot presently be generalized across sexes. Type 2 diabetes was induced by a high-fat diet combined with low-dose STZ [29]. Briefly, mice were fed the high-fat diet (60% kcal from fat) for 4 weeks and then received intraperitoneal injections of STZ (40 mg/kg per day, freshly dissolved in 0.1 mol/L citrate buffer, pH 4.2) for four consecutive days; the high-fat diet was continued thereafter. Fasting (4–6 h) blood glucose was measured from tail-tip blood after STZ injection, and mice with fasting blood glucose ≥ 11.1 mmol/L, accompanied by polydipsia, polyphagia, polyuria, and slow body-weight gain, were regarded as successfully modeled. Twenty-four mice were subjected to STZ induction, and all 24 met the modelling criterion and were randomly assigned to the four diabetic groups (*n* = 6 per group). No animal died or was excluded during the 14-day observation. Wounds were created 7 days after confirmation of sustained hyperglycemia. Fasting blood glucose and body weight were monitored weekly until the end of the experiment (Figure S1). Under isoflurane anesthesia (2–3% in oxygen), the dorsal hair was removed, and a circular full-thickness excisional wound (8 mm in diameter) was created on the dorsum of each mouse with sterile scissors (day 0). The diabetic mice were then randomly divided into four groups (*n* = 6 per group): the diabetic model group (DM, diabetic mice treated with the vehicle), the rhEGF positive control group (rhEGF, diabetic mice treated with 40 IU of rhEGF per wound per day, dissolved in the hydrogel vehicle and applied once daily), the 2% RYR-EH group (diabetic mice treated with the 2% RYR-EH formulation), and the 5% RYR-EH group (diabetic mice treated with the 5% RYR-EH formulation). Non-diabetic mice treated with the vehicle served as the normal control group (NC, *n* = 6). The sample size of *n* = 6 per group was chosen on the basis of previous wound-healing studies using comparable HFD/STZ diabetic mouse models, in which *n* = 6 per group was sufficient to detect a ≥ 20% difference in wound closure rate with approximately 80% power at α = 0.05 [10,28].

The 2% and 5% (*w*/*w*) concentrations refer to freeze-dried RYR-EH powder relative to the total mass of the hydrogel formulation. The concentrations were selected on the basis of preliminary formulation studies: the 2% formulation was the lowest concentration that maintained a uniform dispersion with acceptable spreadability, whereas the 5% formulation approached the practical upper incorporation limit beyond which homogeneity, spreadability, and local tolerability were compromised. A formal pharmacological dose optimisation was not performed and is acknowledged as a limitation. The corresponding formulation (approximately 100 mg per wound) was applied topically to the wound once daily from day 0 to day 14. The wounds were not splinted and were left uncovered; mice were monitored daily for signs of gel removal by grooming, and residual gel was gently wiped away before reapplication. All animal procedures were approved by the Institutional Animal Care and Use Committee of the Third Institute of Oceanography, Ministry of Natural Resources (approval No. TIO-IACUC-04-2025-07-09).

### 4.5. Wound Closure Measurement

Wounds were photographed with a scale bar on days 0, 3, 7, and 14. Before photography and each daily application, residual gel was gently removed and the wound was cleaned with saline. Because the residual coloured preparation could otherwise interfere with image segmentation, wound boundaries were determined from the contrast between the wound bed and the surrounding skin after cleaning, and the same acquisition settings were used for all groups. Wound areas were quantified using ImageJ software (version 1.53, NIH, Bethesda, MD, USA) by an observer blinded to group allocation. The wound closure rate was calculated as follows: wound closure (%) = (A0 − At)/A0 × 100%, where A0 and At denote the wound area on day 0 and at each time point, respectively.

### 4.6. Histological Analysis

On day 14, wound tissues including approximately 2 mm of the wound margin were collected, fixed in 4% paraformaldehyde, embedded in paraffin, and sectioned at 4 μm. Sections were stained with hematoxylin and eosin (H&E) to evaluate re-epithelialization and inflammatory cell infiltration, and with Masson trichrome to quantify the collagen area fraction and assess collagen fiber organization.

### 4.7. Immunohistochemistry and Immunofluorescence

Paraffin sections were subjected to immunohistochemical staining for TNF-α, IL-6, IL-1β, and Ki67, and to immunofluorescence staining for MMP-2, MMP-9, Collagen I, and Collagen III (single staining with DAPI counterstaining) as well as VEGF and CD31 (double staining with DAPI). For immunohistochemistry, antigen retrieval was performed by heating in citrate buffer (pH 6.0), endogenous peroxidase was blocked with 3% (*v*/*v*) H2O2, and sections were incubated with the primary antibody overnight at 4 °C followed by the corresponding secondary antibody and DAB development with hematoxylin counterstaining. For immunofluorescence, fluorophore-conjugated secondary antibodies were applied and nuclei were counterstained with DAPI; negative controls with the primary antibody omitted showed no specific staining. Antibody details (host species, clonality, catalogue number, dilution) are listed in Table S1. VEGF/CD31 double-positive microvessels were defined as structures displaying co-localised VEGF (red) and CD31 (green) fluorescence with a continuous, lumen-like morphology, identified on merged images using ImageJ. Five random fields per section were imaged, and the positive area ratio, integrated optical density, or mean fluorescence intensity was quantified using ImageJ.

### 4.8. Serum ELISA

At the end of the observation period (day 14), blood was collected from the retro-orbital plexus under isoflurane anesthesia, and serum was separated by centrifugation at 3000 rpm for 15 min at 4 °C. Serum levels of TNF-α, IL-6, and IL-1β were determined using commercial ELISA kits according to the manufacturers’ instructions, and absorbance was read with a microplate reader (Multiskan FC; Thermo Fisher Scientific, Waltham, MA, USA).

### 4.9. Statistical Analysis

All data are presented as the mean ± standard deviation (SD). Differences among groups at a single time point were analyzed by one-way ANOVA followed by Tukey’s multiple comparisons test. Wound closure kinetics over time were analyzed by two-way repeated-measures ANOVA with group as the between-subject factor and time as the within-subject factor (with sphericity assumed, as implemented in GraphPad Prism), including evaluation of the group, time, and group × time interaction terms, followed by Tukey’s multiple comparisons test, using GraphPad Prism 9.0 (GraphPad Software, San Diego, CA, USA). All animals completed the study with no missing observations, so a mixed-effects model was not required. Individual animal data points are shown in all graphs. Exact *p* values are reported in the Results, and the compared groups are indicated by the significance brackets in each figure; multiplicity-adjusted *p* < 0.05 was considered statistically significant.

## Data Availability

The data presented in this study are available on request from the corresponding author.

## Supplementary Materials

The following supporting information can be downloaded at: https://www.mdpi.com/article/10.3390/ijms27209020/s1.
