# Supplementary material for: Enzymatic Hydrolysate of Red Yeast Rice Accelerates Diabetic Wound Healing in Mice: Association with Modulation of Inflammatory, Matrix-Remodeling, and Angiogenesis-Related Markers
Source: Int J Mol Sci. 2026 Oct 12;27(20):9020. doi: 10.3390/ijms27209020 (PMC13705068; doi:10.3390/ijms27209020)
Supplement: Supplementary file 1 [file ijms-27-09020-s001.zip › Supplementary Figures.pdf]

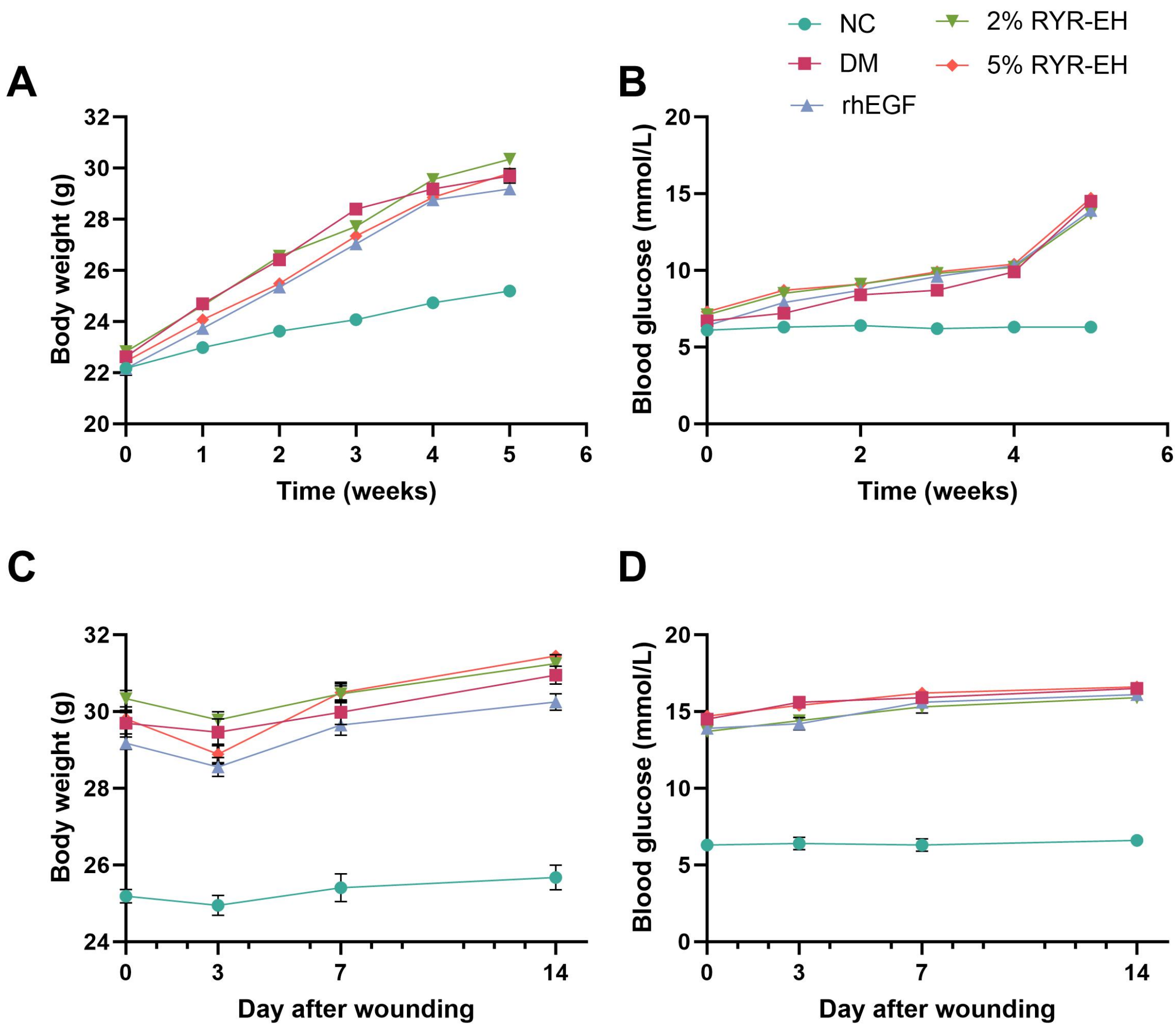

Figure S1. Body weight and fasting blood glucose during modelling and treatment. (A) Body weight during high-fat-diet feeding before diabetes induction; (B) fasting blood glucose during high-fat-diet feeding; (C) body weight during the 14-day wound-healing observation; (D) fasting blood glucose during the observation period. NC: normal control; DM: diabetic model; rhEGF: recombinant human epidermal growth factor positive control; 2% RYR-EH and 5% RYR-EH: wounds treated with the 2% and 5% RYR-EH formulations, respectively. Data are presented as mean ± SD (n = 6).

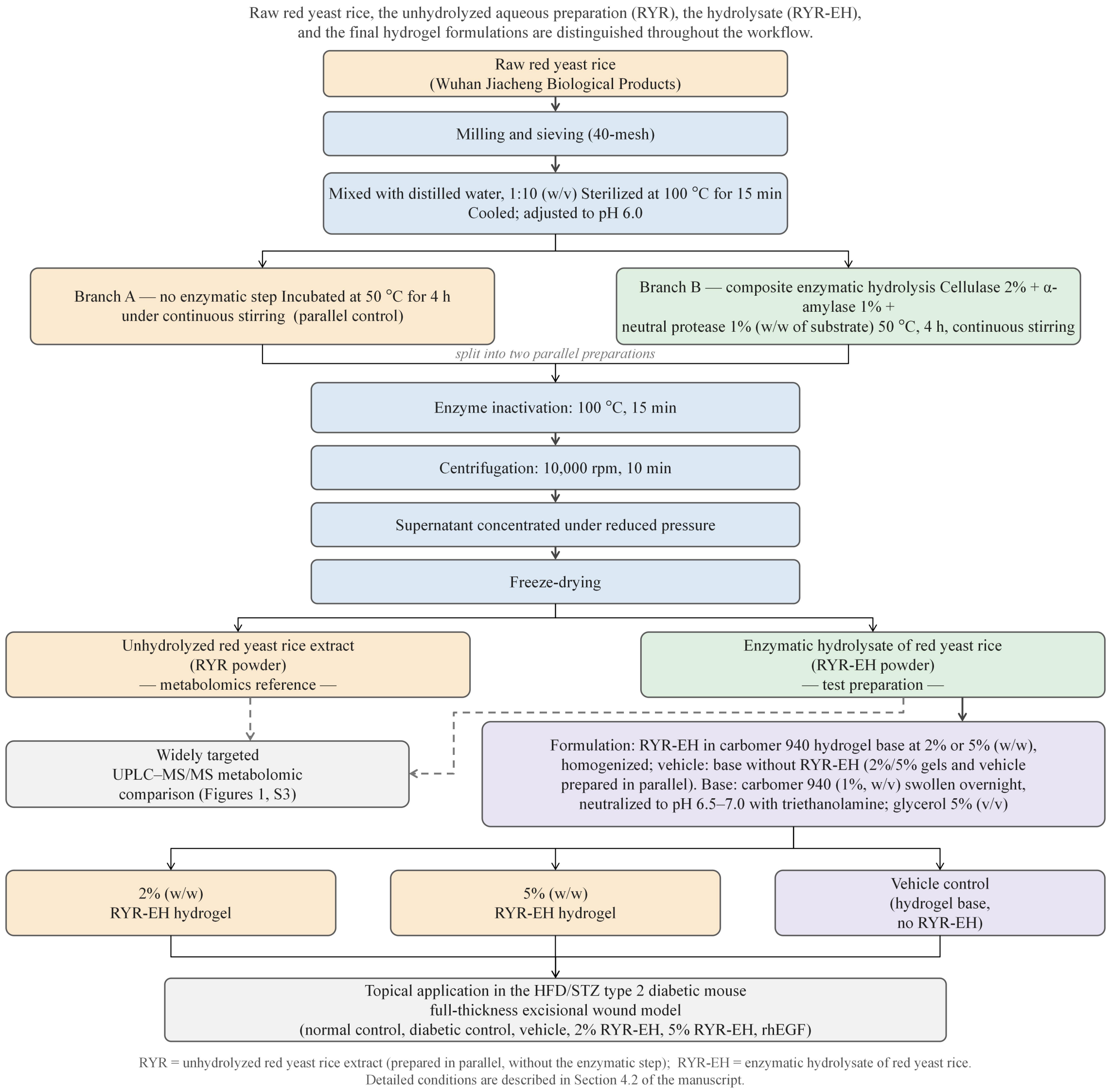

Figure S2. Preparation workflow of RYR, RYR-EH, and the hydrogel formulations.

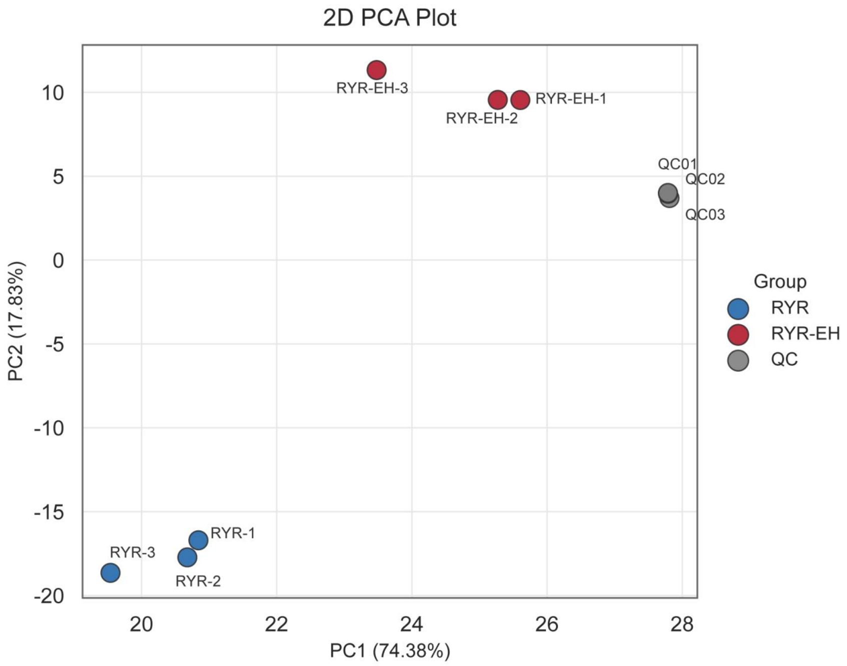

Figure S3. Principal component analysis (PCA) of the widely targeted metabolomic data. The PCA score plot, constructed from log-transformed and Pareto-scaled metabolite intensities, shows clear separation of the unhydrolyzed red yeast rice extract (RYR, n = 3) and the enzymatic hydrolysate (RYR-EH, n = 3) along PC1 (74.38%), whereas the pooled quality-control (QC) samples cluster tightly together, supporting the analytical reproducibility of the profiling.
